# An RF Energy Harvester System Using UHF Micropower CMOS Rectifier Based on a Diode Connected CMOS Transistor

**DOI:** 10.1155/2014/963709

**Published:** 2014-03-17

**Authors:** Mohammad Reza Shokrani, Mojtaba Khoddam, Mohd Nizar B. Hamidon, Noor Ain Kamsani, Fakhrul Zaman Rokhani, Suhaidi Bin Shafie

**Affiliations:** Department of Electrical & Electronics Engineering, Faculty of Engineering, University Putra Malaysia (UPM), 43400 Serdang, Selangor Darul Ehsan, Malaysia

## Abstract

This paper presents a new type diode connected MOS transistor to improve CMOS conventional rectifier's performance in RF energy harvester systems for wireless sensor networks in which the circuits are designed in 0.18 ***μ***m TSMC CMOS technology. The proposed diode connected MOS transistor uses a new bulk connection which leads to reduction in the threshold voltage and leakage current; therefore, it contributes to increment of the rectifier's output voltage, output current, and efficiency when it is well important in the conventional CMOS rectifiers. The design technique for the rectifiers is explained and a matching network has been proposed to increase the sensitivity of the proposed rectifier. Five-stage rectifier with a matching network is proposed based on the optimization. The simulation results shows 18.2% improvement in the efficiency of the rectifier circuit and increase in sensitivity of RF energy harvester circuit. All circuits are designed in 0.18 ***μ***m TSMC CMOS technology.

## 1. Introduction

The growing use of wireless sensor networks (WSNs) and radio frequency identification (RFID) systems has increased the importance of power supply generation for these types of circuits. The use of batteries as the power source for these circuits is usually expensive, inappropriate, and in some cases impossible due to their applications. Therefore, the power supply for these systems is usually generated using the RF energy harvesting method [1–3]. RF energy harvesting operation starts with receiving the existing radio frequency electromagnetic waves in the environment, rectifying the received signal, and converting it into a DC voltage. The generated direct current (DC) voltage is used to supply the required power for WSNs or RFIDs. The generated power using this method should be high enough to provide the required voltage level and current for the subsequent circuits in WSN. On the other hand, the available power for rectification deteriorates further with the reduction in the received energy from the radio waves (usually occurs in farther distances) that decreases the output voltage level and current driving capability of the circuit [4]. Therefore, the output voltage and current in such systems are limited by many parameters, such as available power, input impedance, and rectifier's efficiency. Hence, designing an efficient power supply for these applications has become a real challenge [5–7]. In designing an appropriate system, it is important to design low power circuits and provide an appropriate power supply generation circuit. Generally, a proper power supply to generate the required voltage and current for the subsequent circuit blocks includes the following three main parts: (i) an antenna (ii), a matching network, and (iii) a rectifier circuit.

A micropower rectifier is the most important part in a power supply design for RF energy harvester systems in WSN or RFID applications. The rectifier circuits convert the received RF energy (from the antenna followed by a matching network) to a DC voltage, in order to provide the required power in the WSNs and RFID tags. Today, the design and implementation of an optimized CMOS rectifier having a good sensitivity and an acceptable efficiency has become the most important design bottleneck in supply generation of WSNs and RFIDs (and of RF energy harvesters in general). The previous research on the analysis and efficiency enhancement of CMOS rectifiers can be divided into four categories:

- presenting a mathematical model that matches the fabrication technology of CMOS rectifiers so that the resulting understandings from the model can be used for the design of an efficient harvester system [4–6];
- introducing different circuit techniques to optimize the performance of the conventional CMOS rectifier proposed in [7], which is usually considered as the main core of CMOS rectifier circuits; for example, [8] uses the Schottky diodes in the rectifier circuit to achieve a low threshold voltage, while [9] uses native CMOS transistors with zero *V*_th_ to increase the sensitivity of total rectifiers; the drawback of these methods is that they require a special process to create the Schottky diodes or native low *V*_th  _ transistors, which raises the cost of chip fabrication because the standard CMOS fabrication technologies do not implement Schottky diodes or native CMOS transistors; [10] introduces an internally *V*_th_ cancellation (IVC) technique using the generated output voltage to enhance the input impedance of the rectifier circuit for connection to the matching network and compensation of the threshold voltage of diodes; [11] uses an auxiliary battery along with a battery voltage distributor in order to compensate for the threshold voltage in semipassive applications; meanwhile, [11] uses an auxiliary rectifier chain to compensate for the threshold voltage of the rectifier; all of these mentioned techniques have been useful and could enhance the performance of conventional rectifier circuits;
- introducing new CMOS rectifier structures as the proposed cross-coupled structure in [12] or the proposed self *V*_th_ cancellation (SVC) technique in [12, 13]; these structures could also make reasonable improvements in rectifier circuit fabrication and enhance the performance of conventional rectifier circuits; it is noteworthy that a large number of today's researches are developing these structures;
- introduction of structures that include several transistors (e.g., 3 to 15 transistors) as a diode with close to ideal* I-V* characteristic; using these structures in half-wave and full-wave rectifiers, the performance of the rectifier circuit will be improved due to the enhanced* I-V* characteristic [14, 15].

In designing the optimized CMOS rectifier, it is very important to consider the structure of the diode used in the rectifier and its characteristics. In other words, the threshold voltage and the forward bias current of the diode are the determinants in the rectifier's performance as well as its leakage current in reverse bias region, which play critical roles in the performance of the rectifier circuit. All previous research works had tried to design a circuit with a decreased threshold voltage and leakage current and an increased forward bias current for the rectifying diodes [4–15].

In this paper, a new diode connected transistor having a low “threshold voltage to forward current ratio” (*V*_th_/*I*_*F*_) and a very low leakage current as compared to the conventional diode connected transistors is proposed. The proposed diode is analyzed utilizing simple and accurate models, as in [4–6], and the relevant equations are derived. The* I-V* characteristic of the proposed diode leads to the improvement of the conventional rectifier performance when using the proposed one instead of the conventional one. A design approach to optimize the efficiency of the rectifier using the proposed diode is used as this method presented in [4]. Finally, a 5-stage rectifier circuit is designed and implemented in a 0.18 *μ*m CMOS technology based on the proposed diode and design techniques. Simulation results show a significant enhancement in the performance of the rectifiers using the proposed diode.

## 2. Proposed Diode Structure

**Figure 1:**
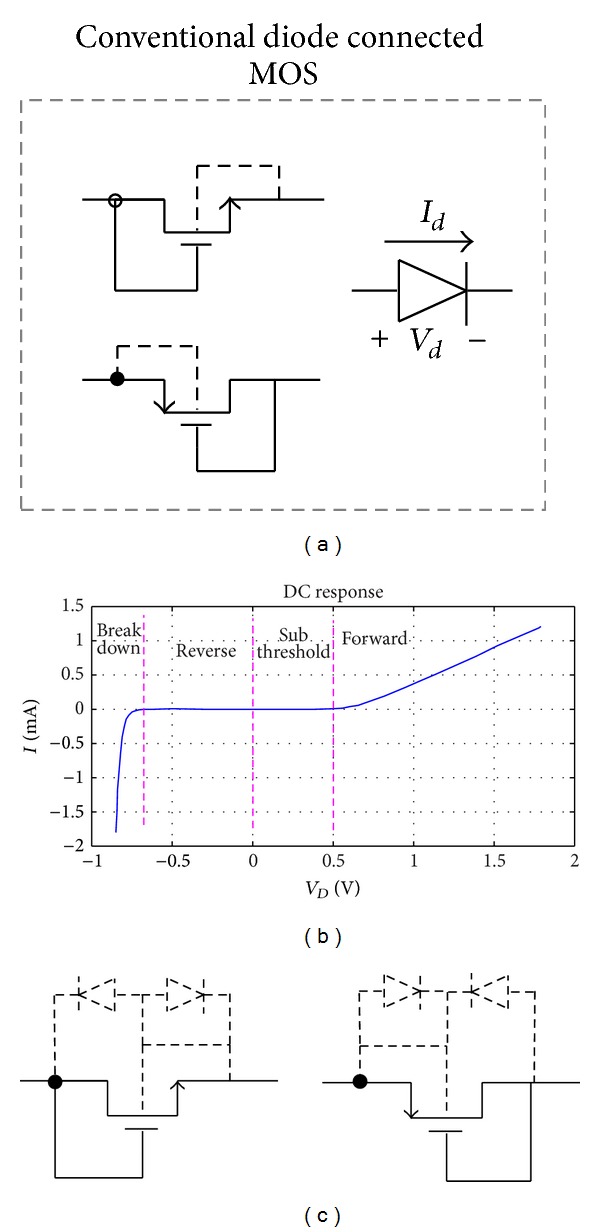
Simulation results for 0.18 *μ*m TSMC process and *W*/*L* = 2 *μ*m/0.18 *μ*m for standard NMOS transistor. (a) A conventional diode connected MOS transistor, (b) its* I-V* characteristic, and (c) the intrinsic PN junction of the MOS transistor.

**Figure 2:**
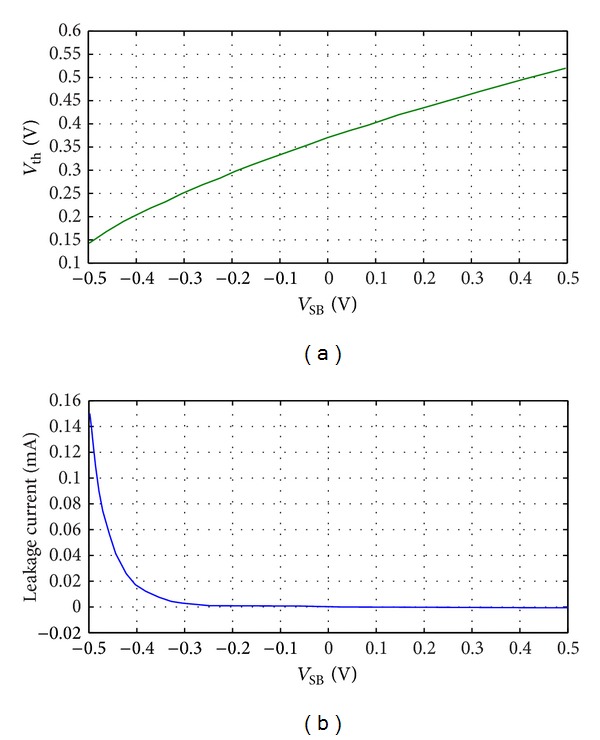
Simulation results for 0.18 *μ*m TSMC process and *W*/*L* = 2 *μ*m/0.18 *μ*m for standard NMOS transistor. (a) Threshold voltage versus source bulk changes and (b) leakage current versus source bulk changes.

**Figure 3:**
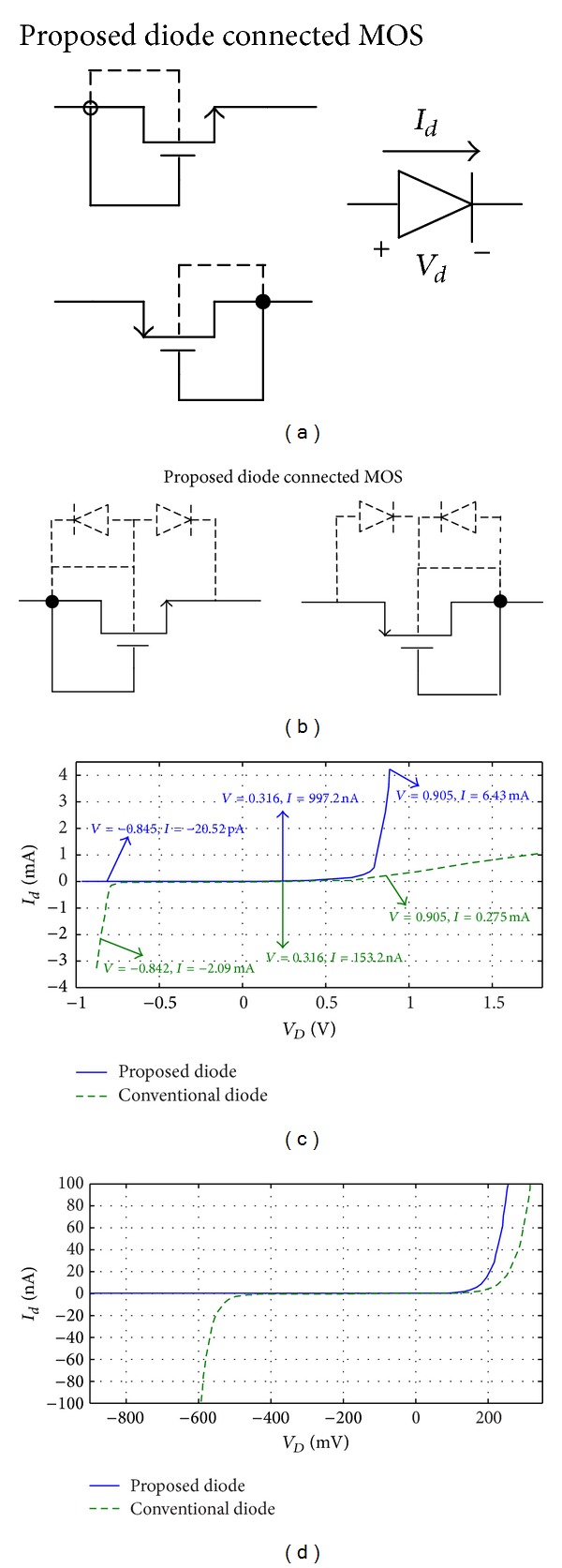
Simulation results for 0.18 *μ*m TSMC process and *W*/*L* = 2 *μ*m/0.18 *μ*m for standard NMOS transistor. (a) The proposed diode connected MOS transistor, (b) the intrinsic PN junctions of the MOS transistor, and (c) the* I-V* characteristic of the proposed diode and (d) zoomed* I-V* characteristic to show difference between forward current and reverse (leakage) current of proposed and conventional diode connected transistor.

The diode connected NMOS or PMOS transistor is the most important part of a rectifier circuit. Therefore, analyzing the* I-V* characteristic of a diode connected transistor helps to truly understand its operation in different regions. Before proposing the diode model it is better to review conventional diode connected transistor's connection schematic, its* I-V* curves, body effect of transistor on its leakage current, and threshold voltage.  Figure 1(a) shows the typical connection of a MOS transistor as a diode. As shown in Figure 1(b), the* I-V* curve can be divided into 4 regions, namely, breakdown, reverse, subthreshold, and forward [6]. In a MOS transistor, in which its bulk is not connected to its source, the body effect can affect the threshold voltage according to

(1) $$\begin{array}{c} V_{\text{th}}=V_{\text{th}0}+k_{1}\left(\sqrt{\varphi_{s}+V_{\text{SB}}}-\sqrt{\varphi_{s}}\right)+k_{2}V_{\text{SB}}, \end{array}$$

where *k*_1_ and *k*_2_ are the parameters which depend on channel doping. *V*_th  _ is the threshold voltage of transistors, *V*_th0_ is intrinsic threshold voltage of transistor, *V*_SB_ is source to bulk voltage of the transistor, and *φ*_*s*_ depends on CMOS technology.  Figure 2(a) shows the variation of threshold voltage of a diode connected transistor versus change of its source to bulk voltage; Figure 2(b) shows the variation of leakage current of a diode connected transistor versus change of its source to bulk voltage.  Figure 2 shows that if there is a positive voltage between the source and bulk of the MOS transistor, the threshold voltage of the diode connected transistor increases and the leakage current of the transistor decreases; however, if there is a negative voltage between the source and bulk of the MOS transistor, the threshold voltage decreased and the leakage current increased. A desirable specification can be defined for an ideal diode in CMOS rectifiers as follows: a proper diode for CMOS rectifier needs a low threshold voltage as well as a very low leakage current in reverse region. A new structure for the diode (diode connected transistor) is shown in Figure 3(a). As shown in this figure, its bulk is connected to the drain instead of the source. Of course there is no difference between the source and drain in CMOS technology, but we propose here that it means that the bulk is connected to the port of the transistor that plays the role of the drain in circuit and is shorted to the gate in diode connection. According to (1), when the proposed diode is biased in the forward region, the source-bulk voltage of the transistor is negative, and in this region, it decreases the threshold voltage of the diode in comparison to the conventional diode connected MOS transistors. This analysis is presented for NMOS diode connected transistor and could be extended for PMOS by reversing the voltages. On the other hand, when the proposed diode is biased in the reverse region, the source-bulk voltage is positive and this leads to reduction in the reverse current of the diode in comparison to conventional ones.  Figure 3(b) shows the intrinsic PN junctions between the bulk and the drain/source of the MOS transistors. As shown in this figure, the intrinsic PN diode in forward bias improves the current of the diode as compared to conventional diode in Figure 1(c), in which the PN diode is reverse biased in the forward region. Conversely, the PN diodes as displayed in Figure 3(b) are off in the reverse bias region in spite of the PN junctions of the conventional diode which are forward biased in this region. So the proposed connection for bulk of the transistors is able to improve the performance of the diode connected MOS transistor for CMOS rectifier design. Figures 3(c) and 3(d) shows the current versus voltage curve of the proposed diode connected transistor in comparison to the conventional diode. As shown in Figure 3(c), in the same *V*_*D*_ of 0.316 V, the conventional diode connected transistor's current is 153.2 nA and the proposed one's current is 997.2 nA. It means 6.2x improvement in diode connected performance. For another example in point of *V*_*D*_ = 0.905 V the conventional diode connected transistor's current is 0.275 mA and the proposed one's current is 6.43 mA. This means 23.38x improvement. For another example in same *V*_*D*_ = −0.842 the conventional one's current is −2.9 mA and the proposed one's current is −20.52 pA. It means 145000000x improvement in reverse region. This huge improvement in reverse region shows that the proposed diode connected transistor does not enter to break down region in compare to conventional one. So according to results of Figure 3 we can conclude that the proposed diode has better performance in* I-V* characteristics in comparison to conventional one.

## 3. N-Stage Rectifier Design Using the Proposed Diode

**Figure 4:**
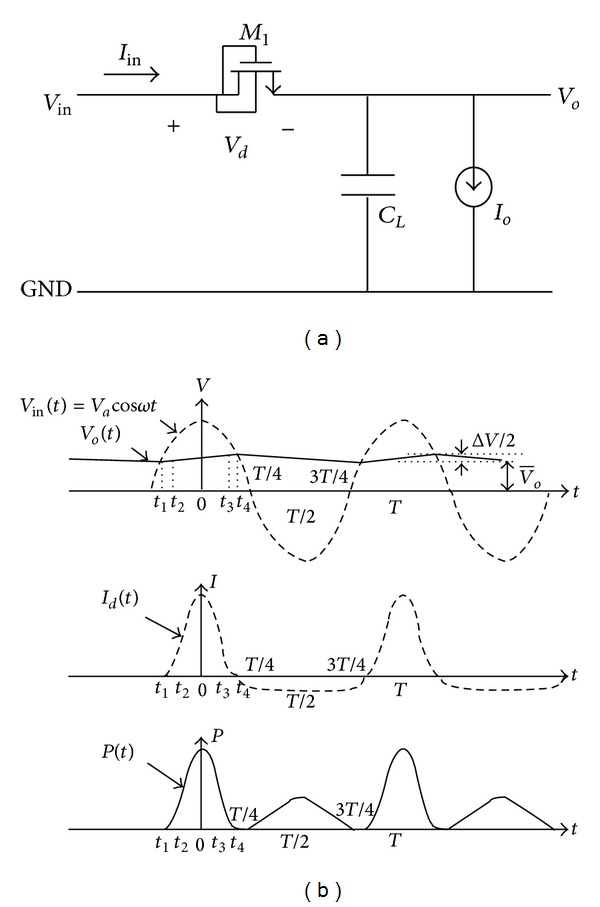
(a) A one-stage rectifier using the proposed diode connected NMOS and (b) its voltage, current, and power waveform.

**Figure 5:**
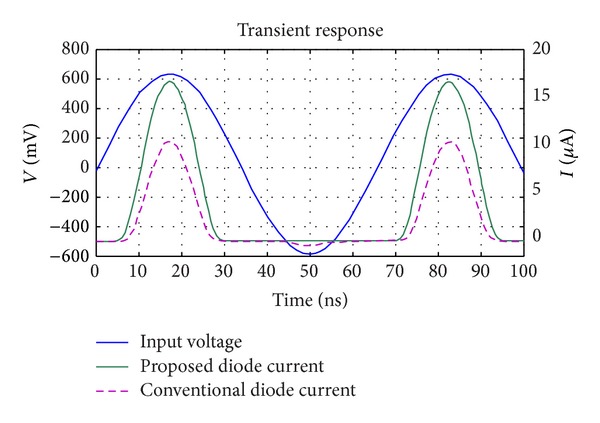
Simulation results for a comparison between the proposed diode current and the conventional one in CMOS rectifiers.

**Figure 6:**
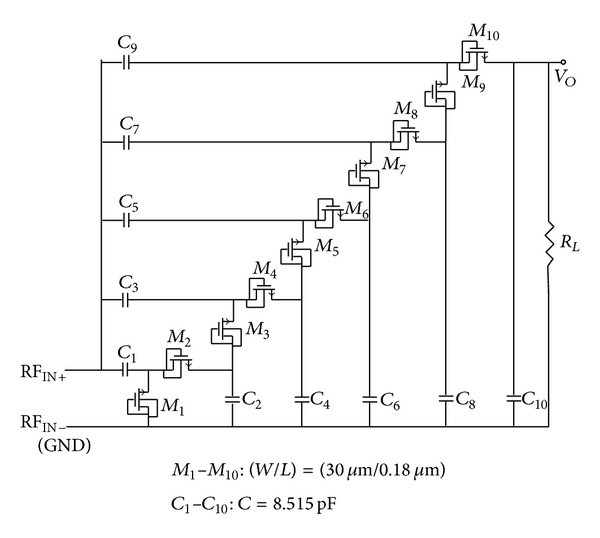
The proposed 5-stage rectifier.

**Figure 7:**
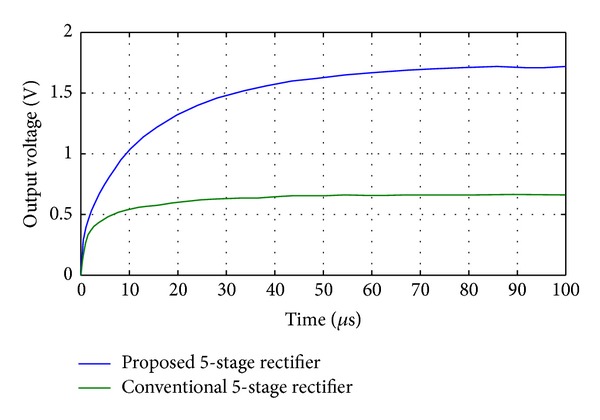
A comparison of the rectified output voltage of a 5-stage CMOS rectifier between the proposed diode connected MOS and the conventional one (by applying the 900 MHz sine wave with 220 mV amplitude).

To design an* N-Stage* rectifier for a specific efficiency, output voltage, and output current, analyzing a one-stage rectifier using the proposed diode is needed.  Figure 4 shows the one-stage rectifier which uses the proposed diode instead of a conventional one. The theory of operation for one stage and N-stage rectifiers can be found in [4, 6] in detail. In this paper, it is shown that if the proposed diode connected MOS transistor is employed in the conventional rectifiers, the output performance will be improved.  Figure 5 shows the simulation result for a full RF signal period for a one-stage rectifier to compare conventional diode and proposed diode operation in the same regions, in which the rectifier operates in forward, subthreshold, and reverse regions. As shown in this figure, the rectifier's output current using the proposed diode is larger (1.7x larger) than the conventional one in the same operating region. On the other hand, the leakage current of the proposed diode in the reverse region is smaller (58x smaller) than the conventional one. These improvements are due to the enhancement in the diode's forward and reverse* I-V* characteristics achieved by the proposed bulk connection. Such improvement is expected from the almost ideal* I-V* characteristic of the proposed diode as displayed in Figure 3.  Figure 6 shows the proposed five-stage rectifier in this work.  Figure 7 shows the rectified output voltage for two similar 5-stage CMOS rectifiers, in which one of them uses the proposed diode connected MOS transistors, while the other one uses the conventional diode connected MOS transistors. As shown in Figure 7, the improvement in output voltage is more than 150% in the transient response of the rectifiers (after 100 uSec past and the output of rectifiers are stable in constant amount).

The design strategy for the CMOS rectifiers can be divided into two categories as follows [4]:

- maximum output voltage with maximum efficiency,
- minimum stages with maximum efficiency.

In this paper to save chip area, method (a) has been chosen. This method has been discussed in [4] in details. As shown in Figure 6, 5-stage rectifier using proposed diode connected transistor with *W*/*L* of 30 *μ*m/180 nm and capacitance of 8.15 pF per stage is the result of using this method for this work's inquiry.

If the input voltage amplitude is large enough (≥400 mV) to turn on the designed rectifier, the efficiency of the proposed rectifier is improved about 50% as compared to the conventional ones. However, in lower input amplitudes, both of the conventional and proposed rectifiers cannot be turned on. The input amplitude of the received RF signal depends on the antenna gain, RF signal frequency, radiated power, and the distance between the RF energy harvester system and the RF signal source. Equation (2) shows the achievable distance as follows:

(2) $$\begin{array}{c} d=\sqrt{\frac{\text{EIRP}\times G_{a}}{P_{a}}}\times \frac{\lambda }{4\pi }. \end{array}$$

In (2), *d* is the distance between the RF energy harvester system's antenna and the source of power, EIRP is the equivalent isotropic radiated power which is limited by local regulations, *G*_*a*_ is the antenna gain, *P*_*a*_ is the received power at antenna, and *λ* is the wave length of the RF signal. To use the proposed rectifier in an RF energy harvesting system, an impedance matching network is required between the antenna and the rectifier to increase the sensitivity of the RF energy harvesting system. Next section proposes a matching network for total energy harvesting system to improve the sensitivity of RF energy harvesting system.

## 4. Design of the Matching Network 

**Figure 8:**
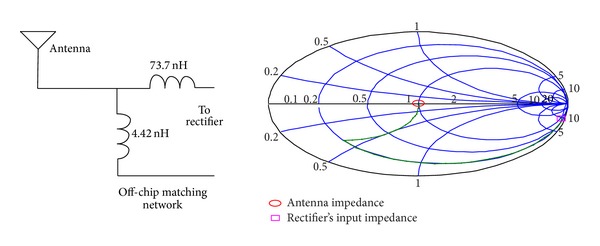
Impedance matching network with off-chip inductors for the proposed rectifier (to be matched to a 50 OHM antenna).

**Figure 9:**
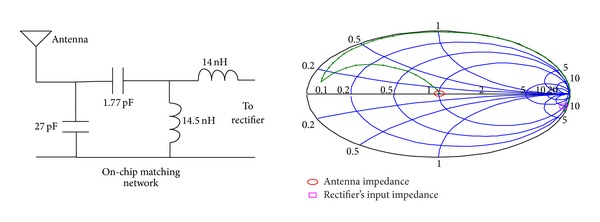
Impedance matching network with on-chip inductors for the proposed rectifier.

**Figure 10:**
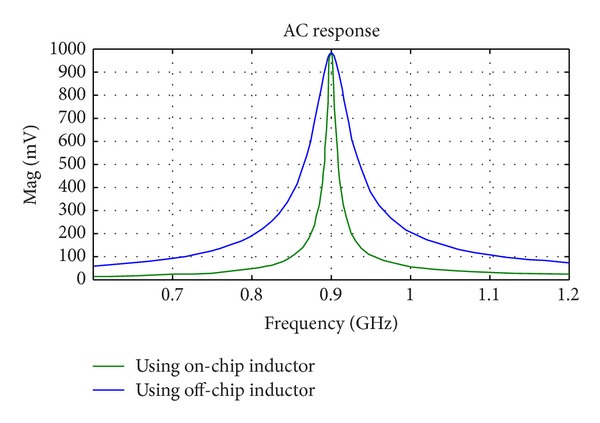
AC response of the input impedance matching network.

**Figure 11:**
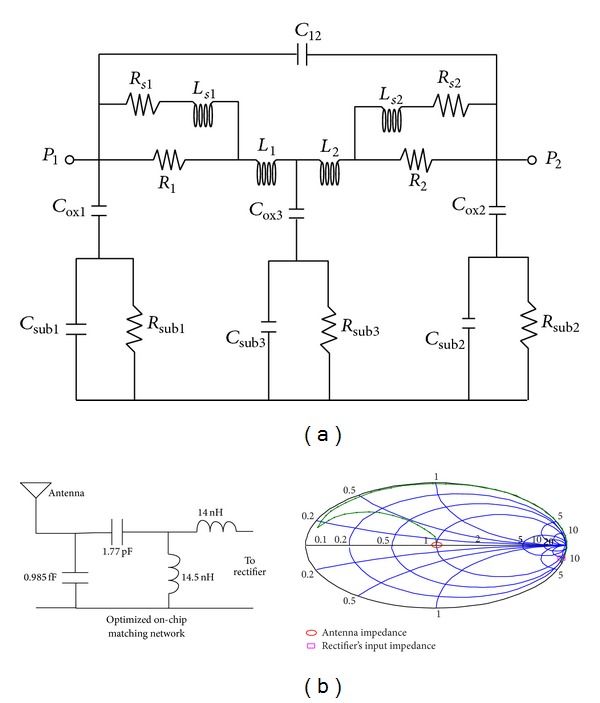
(a) On-chip inductor models in 0.18 *μ*m CMOS technology and (b) optimized impedance matching network for on-chip implementation.

**Figure 12:**
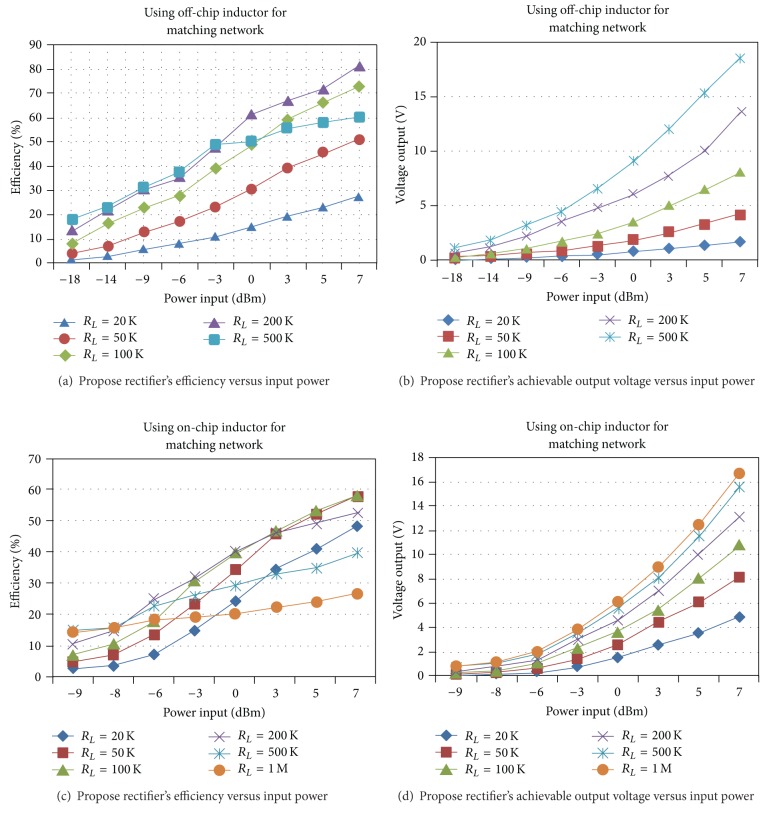
A comparison of the simulation results of the proposed rectifier's efficiency and maximum achievable output voltage versus its input power (there is no voltage limiter at the output).

Impedance matching between the antenna and the rectifier is generally acknowledged as a crucial issue for the optimization of the overall system performance as the impedance matching network can perform a passive amplification of the input voltage [7]. There is no reference method for matching the rectifier because the rectifier is a nonlinear circuit and using the ac analysis is not true. Measuring the input impedance using the harmonic balance method is a good way for calculating the input impedance of the rectifier [16]. For an input amplitude of 430 mV and output load of RL = 1 MΩ, the input impedance is about *Z*_input_ = 9.91 − *j*436.88 for the proposed 5-stage rectifier. Considering a 50 *Ω* antenna, this input impedance should be matched to 50 *Ω*.  Figure 8 shows the matching network for matching *Z*_input_ to 50 Ω in the Smith chart. The calculated impedance of *Z*_input_ can be matched to 50 Ω with only one series inductor of 73.7 nH and a parallel inductor of 4.42 nH. On-chip inductors are usually limited to about 15–20 nH. As shown in Figures 8 and 9, two strategies are used for impedance matching of the proposed rectifier. In the first approach, off-chip inductors are used so that there is no limitation for their inductance value. The second approach uses chip inductors as the matching network components.  Figure 10 shows the ac response of the impedance matching network of the two strategies, respectively. Since the on-chip available inductors are not ideal model and have some parasitic components, the accurate model of the inductor should be considered.  Figure 11(a) shows the inductor model with all of its parasitic components.  Figure 11(b) shows the optimized matching network component which depends on the accurate model of the on-chip inductor model.  Figure 12 shows the output voltage and efficiency of the proposed rectifier versus the input RF power. As shown in this figure, using off-chip inductors improves the sensitivity of the rectifier significantly (−18 dBm), while using the on-chip inductor reduces the rectifier's efficiency. This reduction in sensitivity is due to the limited quality factor of the on-chip inductors in comparison to the off-chip ones. In the next section, the proposed RF energy harvester based on the proposed rectifier and on-chip matching network is presented.

## 5. RF Energy Harvester

**Figure 13:**
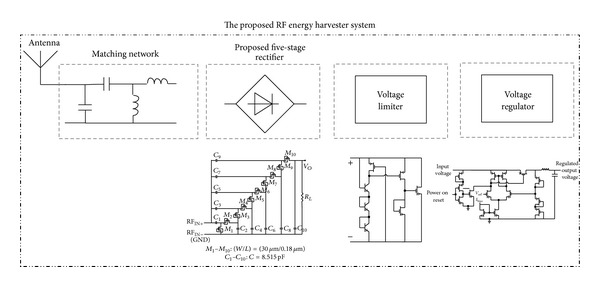
The proposed RF energy harvester system.

**Figure 14:**
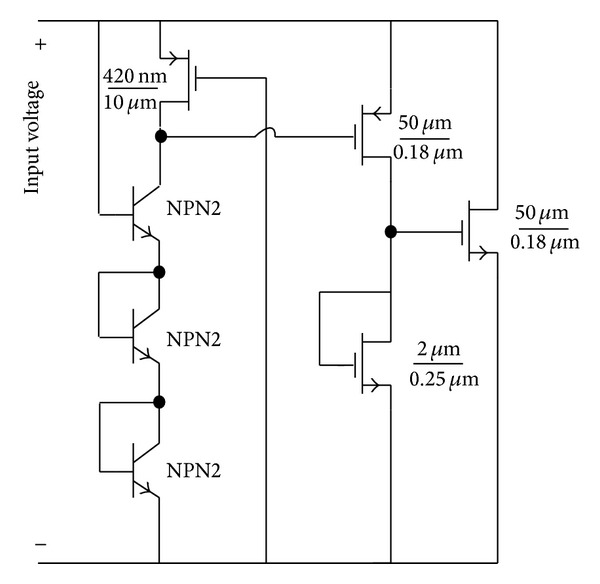
A voltage limiter circuit using at output of the rectifier.

**Figure 15:**
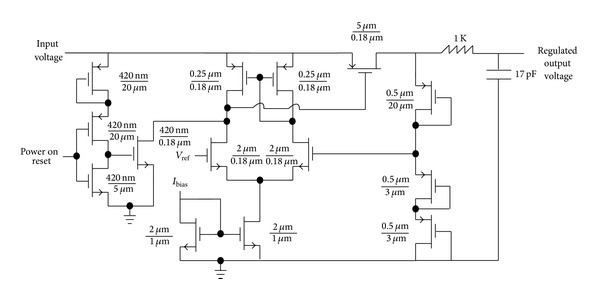
The proposed voltage regulator transistor level circuit topology which is used in the presented RF energy harvesting system for 1.8 V and 1.1 V.

A 5-stage rectifier based on a novel diode connection is proposed in the previous sections with a proper matching network.  Figure 13 shows the proposed RF energy harvester system which uses a matching network, a 5-stage rectifier, a voltage limiter, and a bias block generator, as well as 1.1 V and 1.8 V regulators.  Figure 14 shows the transistor level circuit of the voltage limiter block. This circuit limits the rectifier's output voltage to about 2 volts to prevent the 0.18 *μ*m CMOS transistor from breaking down.  Figure 15 shows the designed voltage regulator for the RF energy harvester system. This circuit is a low drop out (LDO) linear regulator, which gives a fixed output voltage for different input voltages. This variable input voltage is the output voltage of 5-stage rectifier which is limited by limiter. So the input voltage of regulators should be between 1.8 V and 2.1 V.

**Figure 16:**
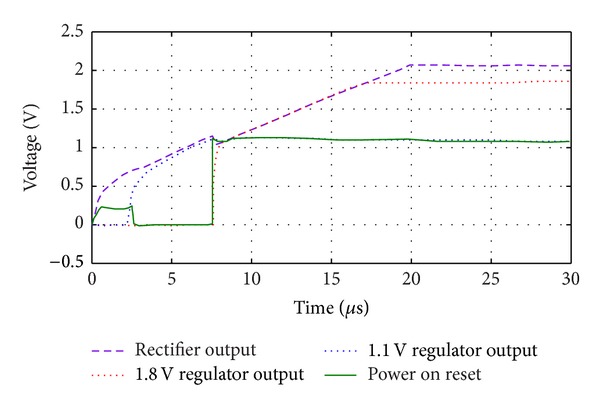
RF energy harvester's startup signals.

Figure 16 shows the proposed RF energy harvester system transient response to a 0 dBm input power at 900 MHz. As shown in Figure 16, firstly, the rectifier's output voltage starts to rise; then the 1.1 V voltage regulator follows it up to 1.1 volt. It remains fixed and regulated at 1.1 volt. The rectifier's output voltage rises up to about 2 volts which is limited by the proposed robust and powerful limiter circuit. The 1.8 volts regulator follows the rectified output voltage after POR rising edge and delivers an accurate 1.8 V output when its output voltage is high enough. This system can be used in each wireless sensor network nodes and RFID tag chips, which can operate with the output voltage and current range of this system.

## 6. Conclusion

A new type diode connected MOS transistor is proposed in this work to improve the diode's* I-V* characteristics in CMOS rectifiers. The proposed diode has better performance in both forward and reverse regions of its operation. It utilizes the intrinsic bulk-source and bulk-drain PN junctions in desired direction to reduce the leakage current and threshold voltage. A one-stage conventional rectifier using the proposed diode is analyzed and compared with the same rectifier using the conventional diodes. Furthermore, an optimization method for maximum efficiency is applied for a design of an optimum CMOS rectifier. Then, a 5-stage rectifier is designed and optimized based on the proposed diode. The rectifier is designed in TSMC 0.18 *μ*m CMOS technology without any additional masks using Cadence. In addition, two matching networks depending on the rectifier input impedance are proposed. Finally, the proposed rectifier is used in a RF energy harvester system in conjunction with other important circuit blocks such as the matching network, over-voltage limiter, and regulators. The simulation shows a very good performance for the proposed rectifier which is comparable with the rectifiers using special process features like the native MOS transistors or the deep submicron CMOS process such as 90 nm. Tables 1 and 2 summarize the comparison between this work and previous works.

## Figures and Tables

**Table 1:** A comparison of this work with previous works.

References	This work	[7]						
CMOS technology node	180 nm	90 nm	250 nm	350 nm	250 nm	300 nm	500 nm	180 nm

Typical threshold voltage	0.51 V	0.45	0.55		0.15	0.53	0.2	0.15

Operating frequency	900 MHz	915 MHz	906 MHz	953 MHz	450 MHz	950 MHz	869 MHz	900 MHz

Additional requirement	Deep n-well	Deep n-well	Precharge phase is needed		Low Vth transistor	Auxiliary battery is needed	Schottky diode	Low Vth transistor

Minimum RF input power	−9 dBm (on-chip inductor) −18 dBm (off-chip inductor)	−18.83 dBm	−17.9 dBm	−9 dBm	−18.6 dBm	−14 dBm	−20.1 dBm	

Rectifier efficiency at minimum RF input power	14.46% (on-chip inductor) 18.08% (off-chip inductor)	11%	9.2%	15.4%	10.4%	1.5%	14.5%	12.6%

Maximum achievable efficiency	58.2% (on-chip inductor) 81.75% (off-chip inductor)							40%

Number of stages	5	24						24

**Table 2:** A comparison of this work with [4].

	Maximum efficiency approach (this work)	Maximum efficiency approach [4]
Number of stages	5	10
Transistor *W*/*L*	30 *μ*m/0.18 *μ*m	30.2 *μ*m/0.5 *μ*m
Output voltage	1.15 V at −8 dBm 50 Ω input power	1.05 V at −8 dBm 50 Ω input power
Capacitor per stage	32 pF/stage (160 pF total)	5.6 pF/stage (56 pF total)
Efficiency at 220 mV V_in_ and 200 K output load	40.17%	33.69%
Additional requirement	Deep n-well	Zero-Vth native transistor
